# Cellular Basis for Response Diversity in the Olfactory Periphery

**DOI:** 10.1371/journal.pone.0034843

**Published:** 2012-04-13

**Authors:** Yuriy Bobkov, Ill Park, Kirill Ukhanov, Jose Principe, Barry Ache

**Affiliations:** 1 Whitney Laboratory, Center for Smell and Taste, and McKnight Brain Institute, University of Florida, Gainesville, Florida, United States of America; 2 Center for Perceptual Systems, University of Texas at Austin, Austin, Texas, United States of America; 3 Department of Electrical and Computer Engineering, University of Florida, Gainesville, Florida, United States of America; 4 Departments of Biology and Neuroscience, University of Florida, Gainesville, Florida, United States of America; Center for Genomic Regulation, Spain

## Abstract

An emerging idea in olfaction is that temporal coding of odor specificity can be intrinsic to the primary olfactory receptor neurons (ORNs). As a first step towards understanding whether lobster ORNs are capable of generating odor-specific temporal activity and what mechanisms underlie any such heterogeneity in discharge pattern, we characterized different patterns of activity in lobster ORNs individually and ensemble using patch-clamp recording and calcium imaging. We demonstrate that lobster ORNs show tonic excitation, tonic inhibition, phaso-tonic excitation, and bursting, and that these patterns are faithfully reflected in the calcium signal. We then demonstrate that the various dynamic patterns of response are inherent in the cells, and that this inherent heterogeneity is largely determined by heterogeneity in the underlying intrinsic conductances.

## Introduction

Attention has long focused on the role of the first olfactory relay, e.g., the arthropod antennal/olfactory lobe and the mammalian olfactory bulb, in generating the spatiotemporal code by which odors are recognized and discriminated (e.g., [1], [2], [3], [4], [5]). However, recent findings indicate that, at least in insects, the primary ORNs themselves are capable of generating temporal activity patterns in an odor- and olfactory receptor (OR)-dependent manner [6], [7], [8], [9], and this input potentially drives the temporal structure of responses seen in the first olfactory relay [6]. These findings shift at least partial responsibility for generating the spatiotemporal code for odors to the olfactory periphery. They also imply that in addition to their primary function of signal detection, amplification, and adaptation, ORNs are capable of producing heterogeneous temporal response patterns with sufficient consistency to be reliably interpreted across the population of ORNs expressing the same OR.

The idea of relying to any extent on a peripherally-generated temporal code raises a number of fundamental questions, the most important of which is how such heterogeneity in discharge pattern is generated and controlled. Nagel and Wilson recently addressed this question using extracellular and local field potential (LFP) recordings from single palp sensilla in insects [8]. They hypothesized that the odor-specific dynamic patterns of response in *Drosophila* ORNs can be largely explained by two elementary consecutive events - transduction and spike generation. Transduction could be reduced to a simple kinetic scheme of ligand-receptor interaction with adaptive feedback control, while spike generation could be described by a differentiating linear filter [8]. While such a reduced, 2-stage model could successfully account for the differences observed in response pattern, it is unclear to what extent the LFP, being an integral measure of receptor activity, reflects the actual dynamics of the underlying receptor current. The LFP could potentially mask patterning contributed by elements downstream of the OR that further shaped the output of the cell. While insects express ionotropic ORs [10], [11], [12], there is controversial evidence for the possible involvement of metabotropic signaling [13], [14], suggesting that the ionotropic OR may possess intrinsic ability to interact with downstream targets in ligand-dependent manner. Support for this idea comes from evidence that some mammalian ionotropic glutamate receptors are now thought to drive second messenger signaling [15], [16], and that some mammalian GPCRs, including ORs, may activate divergent transduction pathways in an ligand-dependent or ligand-biased manner [17], [18], [19], [20], [21].

Here, we explore in more detail how heterogeneity in discharge pattern is generated and controlled in general. We use lobster ORNs, an arthropod model that allows direct patch clamp recording of the receptor current, and one in which there is more compelling evidence for the involvement of metabotropic signaling downstream of ionotropic receptor activation. We demonstrate that several distinct patterns of response can be distinguished among lobster ORNs, including tonic excitation, tonic inhibition, phaso-tonic excitation, and bursting. We show this heterogeneity in responsiveness is reflected in the calcium signal and can be used to determine the ensemble behavior within the population of ORNs. Correlation analysis of calcium signals obtained from populations of ORNs associated with individual olfactory sensilla (aesthetasc) indicates that the response dynamics of the ORNs are likely inherent in the cells. Whole cell voltage-clamp recording shows that the temporal dynamics of the receptor current correlate with the temporal dynamics of the output of the ORN, indicating that the temporal dynamics are largely determined by heterogeneity in the net activating (transduction) current. Finding that different odorants can generate excitatory and inhibitory responses in the same tonic ORN argues this understanding potentially extends to odor-specific temporal activity in ORNs. Our findings collectively argue that the mechanism underlying general heterogeneity in discharge pattern, and likely odor-specific heterogeneity, in lobster ORNs is more complex than can be accounted for strictly by ligand-receptor channel interaction.

## Materials and Methods

### Preparation

Lobster ORNs were studied in an *in situ* preparation developed earlier [22], [23]. Briefly, a single annulus was excised from the olfactory organ (the lateral antennular filament) and the cuticle on the side opposite the olfactory (aesthetasc) sensilla was removed to provide better access to the cell bodies of the approximately 300 ORNs associated with each of the approximately 14 aesthetasc sensilla per annulus. Following treatment with trypsin, papain, or collagenase (1 mg/ml) the ensheathing tissue covering the clusters of ORNs was gently removed to allow access to individual soma. The specimens were mounted on a plastic or glass-bottom 35 mm Petri dish and placed on the stage of inverted microscopes (Axiovert 100, Zeiss, Germany or IX-71, Olympus, Japan). The cell bodies of the ORNs were continuously superfused with *Panulirus* saline (PS) containing (mM): 486 NaCl, 5 KCl, 13.6 CaCl_2_, 9.8 MgCl_2_ and 10 HEPES, pH 7.9. A second superfusion flow of PS allowed odorants to be delivered exclusively to the olfactory sensilla containing the outer dendrites of the ORNs. Both superfusion contours were gravity fed at constant rates of flow.

### Odorants and odorant delivery

**Figure 1:**
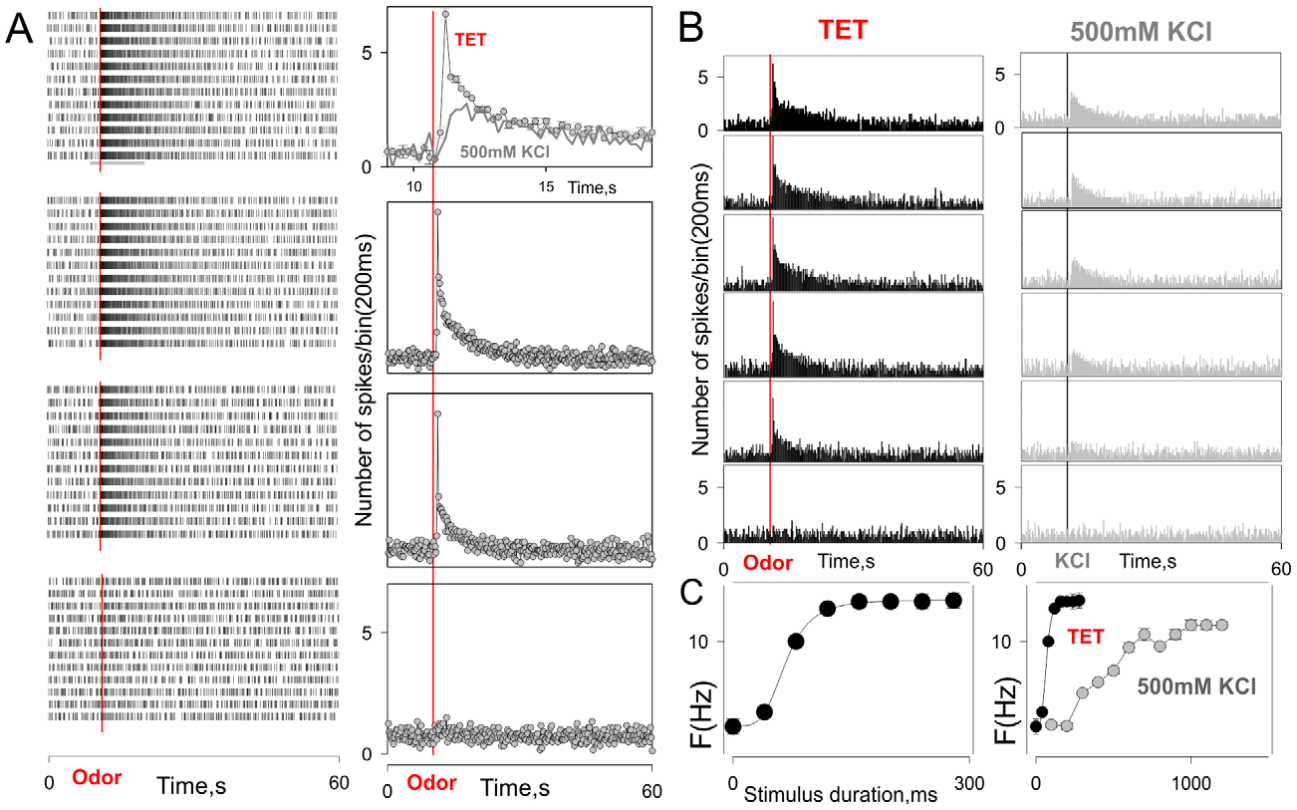
Extracellular responses of individual lobster ORNs in situ. A (left panel) - Raster displays of individual action potentials from a single ORN in response to repetitive stimulation with an odor (within block) at increasing stimulus intensities (between blocks, lowest intensity at bottom) obtained by increasing the duration of the stimulus. A (right panels) - Corresponding peri-stimulus time histograms reflecting the average responses to each stimulus intensity. A (top right panel) – peri-stimulus time histogram shown in higher time resolution within time interval marked by horizontal grey bar (top left). The data are presented as mean±SEM (n = 12). Grey trace is peri-stimulus time histogram showing the same ORN activity evoked by 500 mM KCl applied at the same duration (B). The constancy of the response indicates constancy of the odor intensity profile. B - Peristimulus time histograms of the same cell to different durations of odor stimuli (TET, black colored histograms) compared to cell depolarization with potassium chloride (500 mM KCl, grey colored histograms). Increments in duration for TET were 40 ms (saturation at ∼160 ms), those for KCl were100 ms (saturation at ∼1000 ms). C - Comparison of the dose-response relationships for the odor stimuli (black symbols) and KCl (grey symbols) in B. The nonlinearity of the response to TET re that to K^+^ (A, top right; C, right panel) suggests specific odor amplification. Smooth line - Hill equation approximation with F_max_ = 9; x_1/2_ = 67; h = 4.15; F_b_ = 4. Bin width - 200 ms. To generate the dose-response relationships shown in **C**, mean discharge frequency was determined within 2 sec intervals starting from stimulus onset. The data are presented as the mean±SEM of at least 5 responses. Vertical lines mark stimulus onset. All recordings were obtained from the same ORN.

Unless otherwise noted, the odorant was an aqueous extract of TetraMarine (TET, Tetra Werke, Melle, Germany), a commercially available marine fish food. Flakes of TET were powdered, dissolved in water (0.1 g/ml), filtered through a 0.2 µm syringe filter, and diluted 1∶200 in PS for experiments. The final maximum concentration was ∼0.5 mg/ml. The odorant stream was switched with the flow of PS that otherwise continuously superfused the sensilla (both ∼250 µl/min) using a multi-channel rapid solution changer (RSC-160, Bio-Logic) under software control (Clampex 9, Molecular Devices). Stimulus intensity was controlled by changing the duration of the exposure to the odorant. The initial duration of the odorant pulse, the maximum exposure time, and the interstimulus interval were selected as necessary for different preparations and/or individual ORNs. The maximum exposure time usually did not exceed 1 s, which allowed the odorant to reach its maximal concentration (0.5 mg/ml TET). Importantly, while the approach does not allow precise control of the absolute odorant concentration, it provides highly reproducible stimulus intensity profiles (**Figure 1A, 1B**) and appears to add little if any nonlinearity to changing stimulus intensity (compare TET responses and K^+^ induced ORN depolarization, **Figure 1B**).

### Electrophysiological recording and data analysis

Electrophysiological evaluation of ORN activity was carried out using different approaches. Action potentials (spikes) were recorded from ORNs extracellularly using loose-patch recording. To characterize odor evoked currents of tonically active ORNs we used whole cell voltage clamp recording and whole cell “zero current” voltage mode to record spontaneous burst generation in rhythmically active ORNs. Patch electrodes were pulled from borosilicate capillary glass (Sutter Instrument, BF150-86-10) using a Flaming-Brown micropipette puller (P-87, Sutter Instrument) and filled with PS (extracellular recording). Intracellular solution used in whole cell recordings contained (in mM): KCl180 or 210 mM; NaCl30 or 0 mM; GTP 0.5 mM; ATP 0.5 mM; MgCl2 1 mM; Glucose 696 mM; Hepes 10 mM and Tris-base to adjust pH to 7.8. Resistance of the electrodes was 1–5 mOms as measured in PS. Voltages/currents were measured with an Axopatch 200B patch-clamp amplifier (Axon Instruments) using an AD–DA converter (Digidata 1320A, Molecular devices), low-pass filtered at 5 kHz, sampled at 5–20 kHz. Data were collected and analyzed with pCLAMP 9.2 software (Molecular Devices) in combination with SigmaPlot 10.0 (SPSS). When necessary, multiunit recordings sampled usually at 20 kHz were sorted into individual unit recordings using the template search procedure provided in pCLAMP 9.0 software. The time of occurrence of the spike was taken as the time of peak current deflection, i.e., the peak of the spike.

### Calcium imaging and data analysis

After enzymatic treatment and cleaning, the olfactory antennular segments were placed in an Eppendorf tube in PS containing the fluorescent calcium indicator of choice (Fluo-4AM or Fura-2AM) at 5–10 µM prepared with 0.2–0.06% Pluronic F-127 (Invitrogen). Fura-2AM was used to estimate the absolute calcium concentration (see [23] for details). The tube was shaken for about 1 hr on an orbital shaker (∼70 rpm). The tissue was transferred into fresh PS and mounted for imaging. After the dye loading the preparation typically remained viable for 2–4 hr. Fluorescence imaging was performed on an inverted microscope (Olympus IX-71) equipped with a cooled CCD camera (ORCA R2, Hamamatsu) under the control of Imaging Workbench 6 software (INDEC Systems). The software allows for complete integration with electrophysiological recording using Clampex 9, including triggering of the imaging system and synchronization of both optical and electrical signal acquisition. A standard FITC filter set (excitation at 510 nm, emission at 530 nm) was used for single-wavelength measurements. Ratiometric imaging was performed using a Fura-2 filter set (excitation at 340 nm or 380 nm, emission at 510 nm). Images were collected usually at the rate ∼3, 5 or 24 Hz (specified in the figure legends). Recorded data were stored as image stacks, analyzed off-line using Imaging Workbench 6 or ImageJ 1.42 (available in the public domain at http://rsbweb.nih.gov/ij/index.html).

Numerical analysis including event analysis and kinetic parameter estimates was performed using either ClampFit 9 (Molecular Devices), or SigmaPlot 10 (Systat). For calcium oscillation analysis the recordings were first compensated for any slow drift in fluorescent intensity and the threshold based event detection algorithm (ClampFit) was applied to identify individual oscillatory events. The time of occurrence of the oscillation was taken as the time of the oscillation peak. Inter-event intervals were taken as the time between the peaks of two subsequent events. The oscillation period for rhythmically active ORNs was estimated as the mean of a Gaussian approximation to the inter-event interval distribution. The data are presented as the mean±SEM of n observations unless otherwise noted. In total, data were obtained from 31 lobsters. All recordings were performed at room temperature.

Correlation analysis of the calcium signals of ORN ensembles was performed using original algorithms developed in Matlab (Mathworks, Inc). A burst-triggered averaging method was used for the detection of correlated activity for its simplicity to account for non-stationarity of the calcium signal. 1000 jittered surrogates of burst triggered average was generated by jittering each burst time with a 4 sec standard deviation Gaussian distribution. The surrogates were used to estimate the null hypothesis. This procedure is effective when the non-stationarity is slowly changing (over a few sec) relative to the rapid temporal features of neural activity. Z-scores were plotted without adjusting for multiple comparisons.

## Results and Discussion

### Lobster ORNs collectively show different patterns of discharge

**Figure 2:**
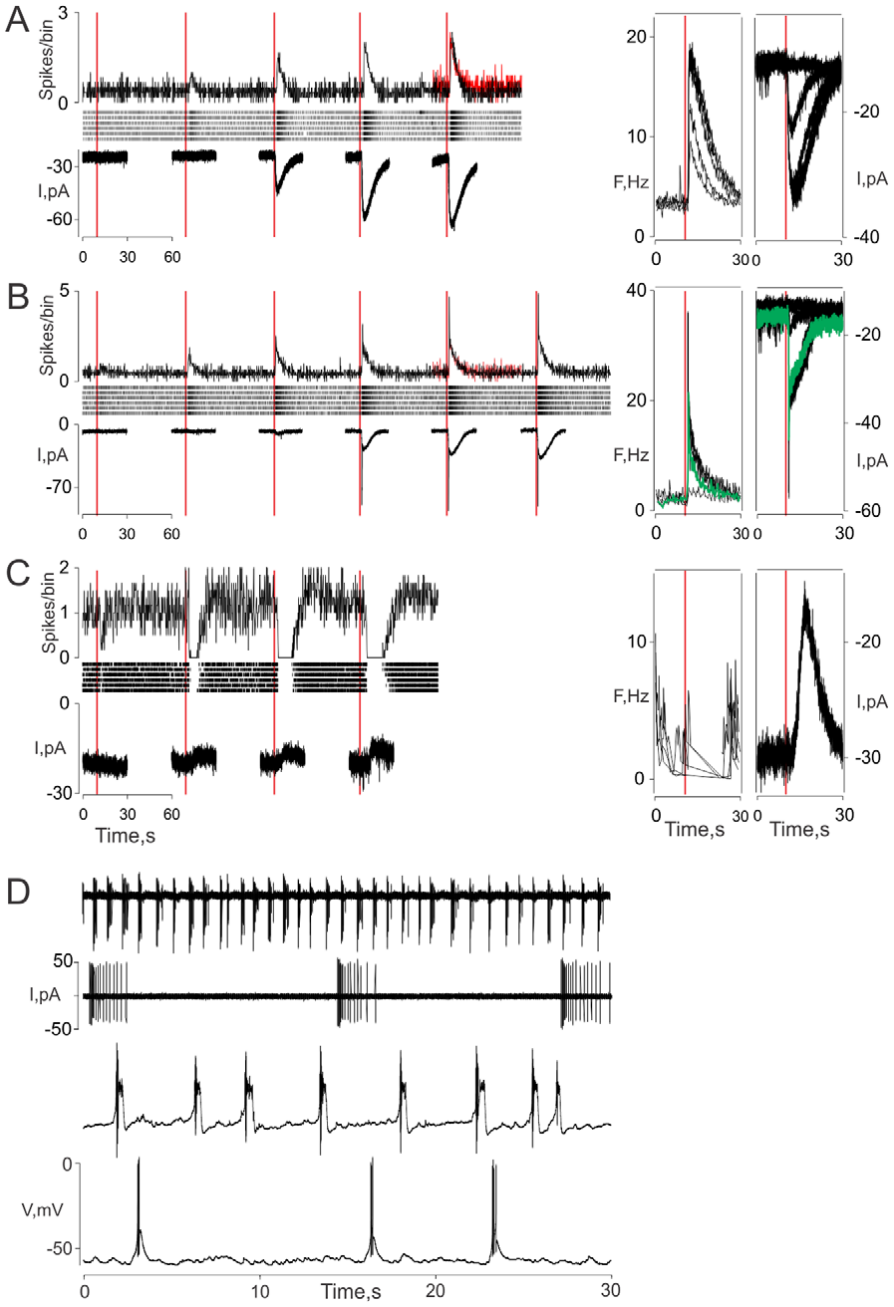
Basic patterns of spontaneous and odor evoked activity of lobster ORNs characterized electrophysiologically. A – tonically active ORNs gradually increase their ongoing rate of discharge (top) or whole-cell inward currents (bottom) in a stimulus intensity dependent manner to odorants. B – phaso-tonic ORNs are characterized by distinctive transient excitatory constituent of the ORN responses followed by slow, more linear component kinetically similar to the responses observed for tonically active ORNs (compare with A). C – some tonic ORNs respond to odor stimulus with inhibitory response patterns in stimulus intensity dependent manner. Histamine (400 µM – maximal concentration, C, left plots) and TET (C, right panels) were used as stimuli. Top panels - Extracellular recording from individual lobster ORNs. Raster displays of individual action potentials from a single ORN in response to repetitive stimulation with an odor at increasing intensity (from left to right, 6 trials shown). Left top panel – corresponding peristimulus time histograms showing average responses. Left bottom panels – whole-cell recordings. Note, extracellular and whole cell recordings (left panels) were obtained from different ORNs. Right plots – extracellular activity and whole cell currents recorded from the same ORNs. ORN discharge activity is expressed as instantaneous spike frequency (F,Hz). Green traces (B, right panels) show ORN responses to intermediate intensities of the stimulus. D – Spontaneous activity of rhythmically active ORNs. Bursting ORNs with different intrinsic bursting frequencies recorded extracellularly (two top recordings) and using whole-cell patch clamp recording (two bottom traces). Experimental conditions: Extracellular recordings were obtained using loose cell-attached patch recording with standard patch clamp electrode filled with PS. Whole-cell recordings, intracellular solution (mM): KCl 210, Glucose 696, EGTA 1; Hepes 10; pH 7.8 (A, B); KAcetate 180, NaCl 30, Glucose 696, EGTA 1, Hepes 10 and pH 7.8 (C); KCl 180, NaCl 30, GTP 0.5, ATP 0.5, MgCl_2_ 1, Glucose 696, EGTA 1, Hepes 10 and pH adjusted to 7.8 (D); mode – voltage clamp recording (A, B, C), zero-current voltage recording (D). Holding potential was −70 mV (A, B, C), −50 mV (C, left bottom traces). Bin width – 200 ms. Red peristimulus time histograms in A, B represent ORN activity evoked by 500 mM KCl applied at the same duration (like in the **Figure 1B**). Note again, the nonlinearity of the ORN responses observed in particularly in B suggests involvement of an amplifying mechanism/s.

As reported earlier [24], lobster ORNs fall into two global subpopulations that differ in their spontaneous activity and pattern of spiking: tonically active cells that generate spikes at different frequencies (**Figure 2A**), and rhythmically active cells that generate bursts of spikes at different frequencies (**Figure 2D**). Our data suggest the tonically active cells to be further divided into at least three distinguishable groups based on their pattern of odor response. (1) Tonic excitatory neurons (∼34%) linearly follow the time course of the stimulus (**Figure 2A**). Their spontaneous frequency ranges from 0.01 to 8.25 Hz with a mean discharge of 2.4±0.2 Hz, n = 87. While the amplitude of the responses proportionally reflects the stimulus intensity, the response pattern does not significantly change with stimulus intensity. (2) Phaso-tonic neurons, (∼62%) respond with a distinct transient peak that varies in amplitude from cell to cell and stimulus intensity (**Figure 2B**). The transient component is followed by a slow linear component kinetically similar to the responses observed for tonic cells. The spontaneous frequency of these ORNs ranges from 0.01 to 8.3 Hz, with a mean discharge of 2.3±0.13 Hz, n = 160. (3) Tonic neurons with inhibitory responses (∼4%) show a decrease in spontaneous activity (range, 1.0 to 8.7 Hz, mean – 3.6±0.9 Hz, n = 10) in response to complex odor stimulation. The percentage of the ORNs characterized by inhibitory response patterns increased when the ORNs were stimulated by some single compounds (e.g. histamine, 10–40 µM, ∼30%, 6 out of 17 cells, **Figure 2C**). The inhibitory response is accompanied by a hyperpolarization in a concentration-dependent manner (**Figure 2C**), although it is unclear whether this is the result of a hyperpolarizing outward current or inhibition of an excitatory conductance that is partially active at rest. While our previous findings suggest that histamine may directly activate chloride channels located on the ORN somata [25], typicaly the activity of the histamine gated chloride channels, including those expressed in lobster ORNs, is characterized by profound inactivation and desensitization (personal observation, [25]). Thus histamine acting here as an inhibitory odorant presumably is through a different mechanism than in the case of the ligand-gated chloride channel described previously.

As mentioned above some lobster ORNs are inherently rhythmically active. **Figure 2D** shows the spontaneous activity of these ORNs recorded extracellularly (top panels, two ORNs with different bursting frequencies and burst patterns) and in whole-cell (“zero current”) voltage mode (bottom panels, two ORNs with different bursting parameters). Brief odor stimuli elicit bursts in these ORNs in a phase-dependent manner [24]. Since the coding of different parameters of odor stimuli based on pacemaker-like neurons is a complex phenomenon, we will not consider it here other than note that the information conveyed by rhythmically active neurons is presumably qualitatively different from that conveyed by graded trains of action potentials and is inherent in ensemble of cells, not individual ORNs.

### The pattern of discharge reflects the dynamics of the underlying receptor current

Under whole-cell, voltage-clamp (holding potential set between −70 and −60 mV), odorants activated either excitatory inward or inhibitory outward currents (**Figure 2A, 2B, 2C**, left panels). As with the odor-evoked activity patterns recorded extracellularly, three basic response patterns could be observed: tonic (95/174 cells tested, ∼55%, Figure 2A), phaso-tonic (∼39%, **Figure 2B**), and inhibitory (∼6% when stimulated with TET) suggesting that the discharge pattern potentially correlates with the shape of the transduction current. Recording extracellularly and in the whole-cell mode from the same ORN not only support this idea, but indicate that the pattern of discharge closely reflects the dynamics of the underlying whole-cell receptor current (**Figure 2A, 2B, 2C**, right panels) and does not require a significant contribution from downstream conductances (e.g., voltage-gated channels generating action potentials).

While the detailed mechanism of response generation in lobster ORNs is still unclear, the input current is driven by iGluRs [26], [27] that are orthologs of insect IRs and form heteromeric ionotropic receptor-channels [11], [12]. In lobster ORNs, however, there is long standing evidence for the involvement of metabotropic signaling in olfactory transduction [28], [29], implying that lobster IRs are capable of driving metabotropic signaling. Indeed, the fast phasic component seen in the response of some lobster ORNs suggests amplification possibly mediated by metabotropic signaling (**Figure 1B** **,** **2B**). The idea that ionotropic receptors drive metabotropic signaling in lobster ORNs would be consistent with recent evidence that insect ORs can activate G proteins *in vitro* [14] and can activate PLC *in vivo* [30], suggesting that insect ORs, also ionotropic receptors [10], [13], retain the capacity to couple to second messenger signaling through G proteins. It would also be consistent with recent evidence that insect gustatory receptors (GRs), generally held to be traditional GPCRs, can also function as ionotropic receptor-channels [31]. Thus, emerging evidence in insects is consistent with the present findings in lobster that the mechanism underlying heterogeneity in discharge pattern, and likely odor-specific heterogeneity, is more complex than can be accounted for strictly by ligand-ionotropic receptor channel interaction, although more work clearly is needed to resolve this important question.

### The calcium signal reflects the electrophysiological patterning and suggests independence of ORN activity

**Figure 3:**
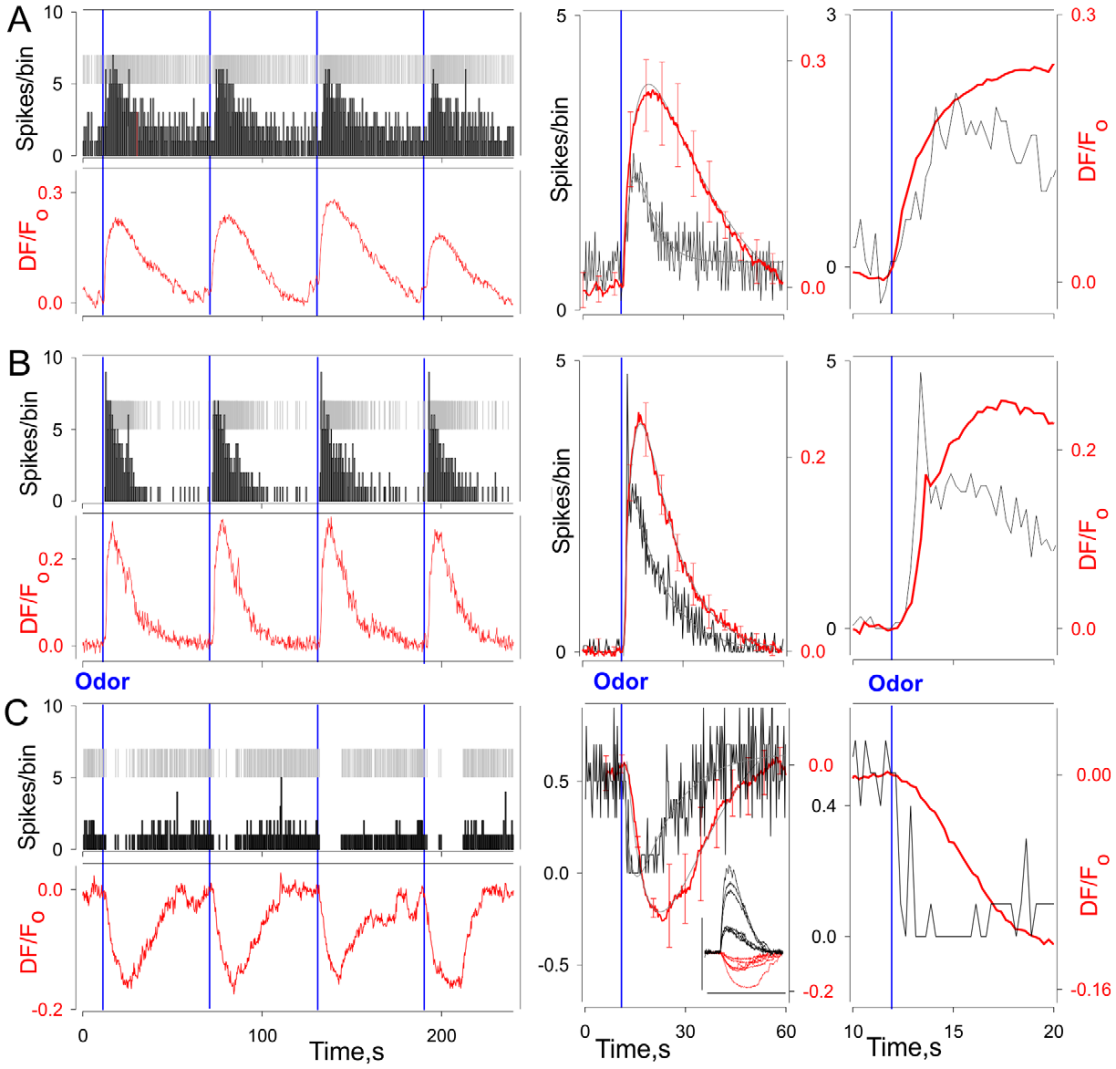
Calcium imaging of the basic lobster ORN types. Simultaneous spike (top) and calcium signal (bottom) recordings from tonically active ORNs (with excitatory tonic (A), phasotonic (B) and inhibitory (C) response patterns). Tonically active ORNs were repeatedly stimulated by the same stimulus intensity (four responses are shown for each ORN types) and both spike and calcium signal responses were then averaged (middle and right panels). Right plots – same as middle in higher time resolution. Note, characteristic shape of the calcium response allows reliable distinguishing phasotonic cells. TET (A,B) and Histamine (400 µM – maximal concentration, C) were used as stimuli. C, middle panel inset shows calcium signals from six ORNs demonstrating both excitatory (upward traces) and inhibitory (downward traces) response patterns when stimulated by TET and Histamine respectively. Bin width – 0.5 s (left panels), 0.25 s (right panels).

To determine whether the calcium response reflects the dynamics of the various types of electrophysiological responses, we recorded the electrophysiological activity of individual ORNs simultaneously with calcium imaging and averaged and compared the resulting calcium signal with the spike discharge. The calcium response reflected the dynamics of ALL the three patterns of tonic response (**Figure 3**). The polarity of the calcium response (increase vs decrease of [Ca^2+^]_i_) allowed unequivocal identification of ORNs with inhibitory responses (**Figure 3A** **vs 3C**). Note that different stimuli could elicit responses of different polarity within the same ORN (**Figure 3C**, middle, inset). The ascending phase of the phaso-tonic responses generated a characteristic “brakepoint” in the calcium signal that allowed these responses to be distinguished from the monotonic response of tonic ORNs (**Figure 3A** **vs 3B**, right panels).

**Figure 4:**
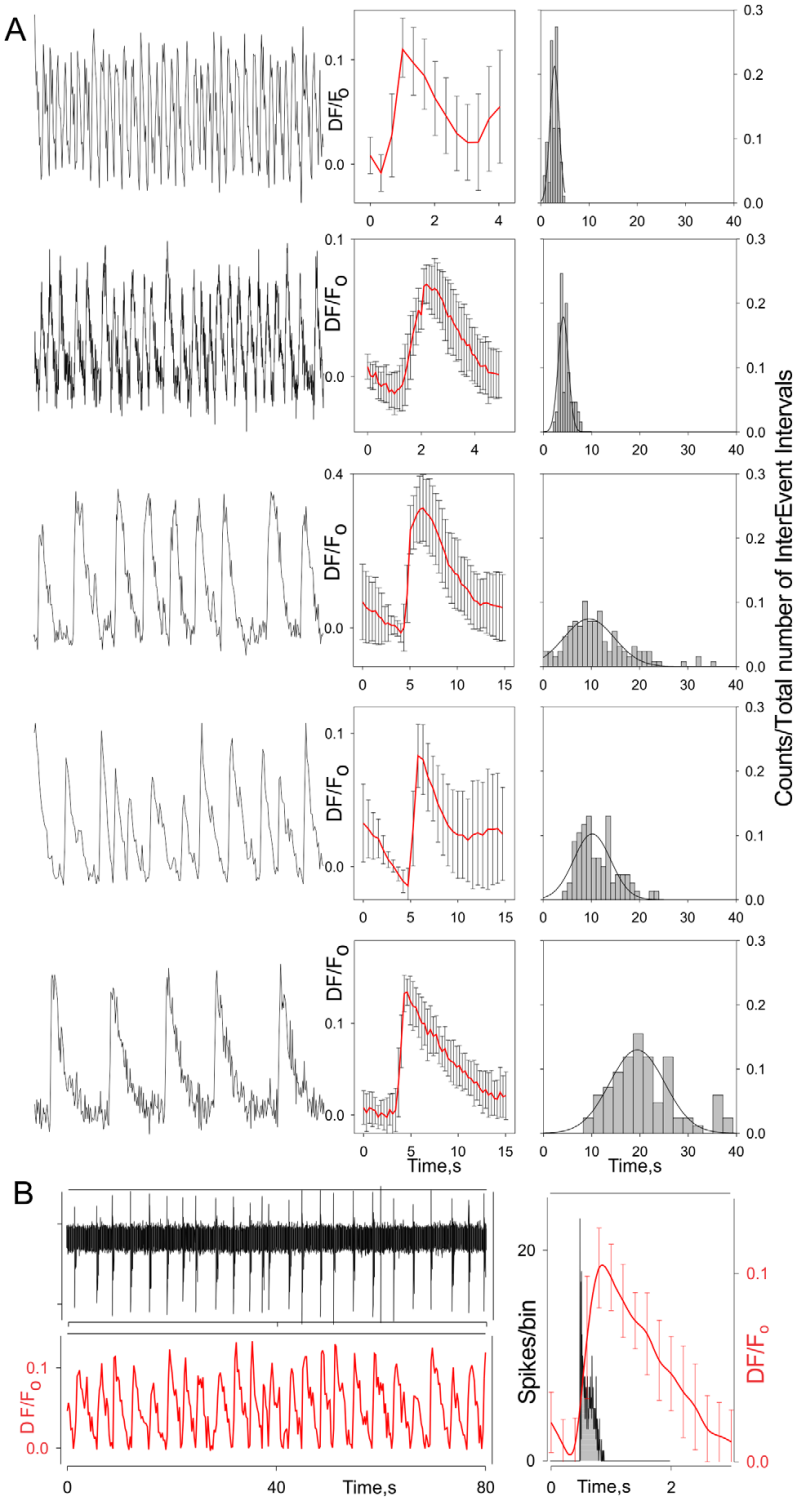
Calcium oscillations in the bursting ORNs. **A** - Each ORN is characterized by its own intrinsic oscillatory/bursting frequency (left, right) and oscillation pattern (middle). To generate averaged calcium oscillation patterns oscillatory cycles were aligned relative to the peak of oscillation cycle. Bars show standard deviation of some average values of the calcium signal. Inter -event/-oscillation interval histograms were normalized to the total number of events included in the analysis and approximated by Gaussian distributions (right panels, smooth lines) to yield following individual bursting ORN frequencies (Hz, from top to bottom): 0.36, 0.24, 0.11, 0.09 and 0.05. Time of a peak oscillation was taken as an event time. B - Simultaneous spike (left top) and calcium signal (left bottom) recordings from bursting ORN. Right panel - both burst related inter spike interval histogram and averaged calcium signal oscillation were aligned relatively to the time of the first spike in a burst. Burst parameters (B, right): number of spikes per burst −15.1±0.4; interburst interval −2.9±0.1 s; burst duration – 0.3±0.007 s. Error bars are standard deviations shown for some average values. Bin width - 0.5 s, 1 s, 2 s (A, right), and 0.005 s (B, right). Slow changes in calcium signal levels mainly caused by calcium indicator bleaching were manually subtracted.

The calcium signal also reflected the dynamics of the bursting ORNs. Spontaneous calcium oscillations had an average peak amplitude of 214±17 nM (n = 17 cells). We assume that these calcium oscillations reflect the spontaneous activity of bursting ORNs since (1) while individual cells differ in their patterns of oscillations, the oscillatory pattern for any given cell is consistent (**Figure 4A**, left, middle), (2) the frequency of the calcium oscillations for any given cell is consistent even though it differs for different cells (**Figure 4A**, left, right), (3) the oscillating neurons co-localize in the same cluster with those demonstrating steady state calcium signals (**Figure 4**, see also [23]) and (4) the overall distribution of the calcium oscillation frequencies is in good agreement with the bursting distribution frequencies (0.16±0.01 Hz, ranged from 0.015 to 0.9 Hz, n = 84, compare with [24]). Indeed, we could show this directly by combining calcium imaging of individual oscillating ORNs with extracellular electrophysiological recording from the same cells. As shown for one cell in **Figure 4B**, each burst is accompanied by a robust calcium oscillation. Comparison of calcium oscillations and bursting dynamics suggests that burst generation appears earlier in the calcium oscillation cycle and perhaps even triggers calcium increase (**Figure 4B**, right). Thus, the calcium signal allows characterizing the global output of lobster ORNs in the context of differences in the activity patterns of individual ORNs.

**Figure 5:**
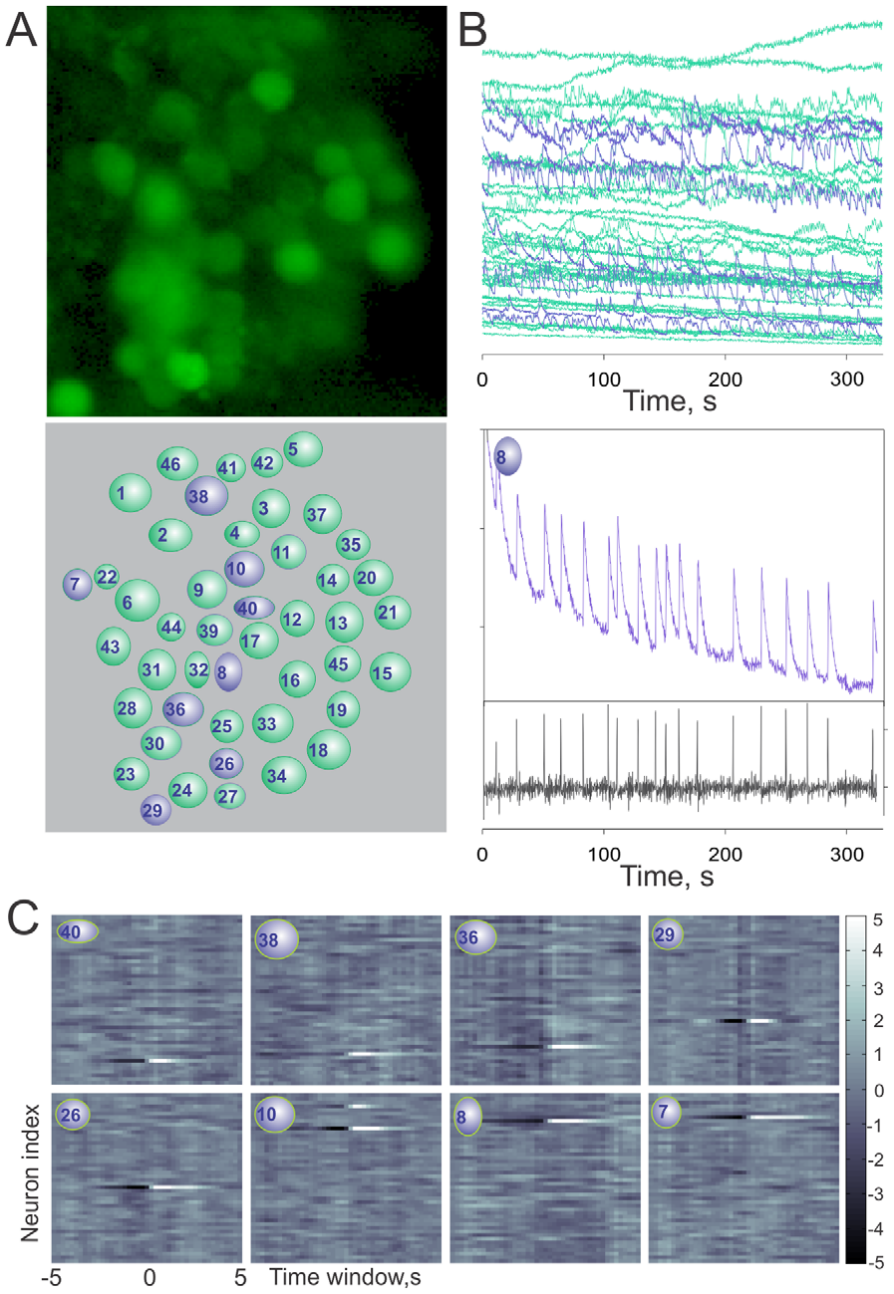
An example of correlation analysis. ORN ensemble spontaneous activity forming single neuronal cluster (A) was analyzed relative to that of the same cluster individual rhythmically active ORNs (A, bottom panel, blue circles). Individual cell regions analyzed were carefully selected to avoid overlapping of optical signals (A, bottom). The raw calcium signal intensity time series (B) was filtered to reduce common noise and normalized. Burst-related calcium oscillations were detected by threshold-based searching the first numerical derivative of the signal (B, bottom panel, vertical bars indicate the presumptive burst/oscillation timing). Reverse correlation analysis was used to test whether, and to what extent, the spontaneous activity of the bursting ORNs could modulate activity of ORNs within the same neuronal cluster. We detected the bursting activity and averaged the time-locked calcium signal intensity within a 5 second window. If a repeated activation or deactivation occurs, averaging would effectively reduce the noise and reveal the interaction. Adjacent ORNs 10 and 4 demonstrate “correlated activity” likely reflecting of overlapped optical signals from the ORNs. Significance of the correlation can be verified with a non-stationarity corrected z-test (C, p≤0.01 if |z|>2.58, p≤0.001 if |z|>3.29).

To confirm the general assumption that receptor current underlying the activity patterning is inherent in the ORNs and does not reflect interaction between or among ORNs, we carried out correlation analysis of the spontaneous activity of ORNs in the same clusters, i.e., those with the greatest potential to interact, relative to that of individual rhythmically active ORNs (**Figure 5A, 5B**). Neuronal clusters and individual cell regions analyzed were carefully selected to avoid significant overlapping of optical signals. The raw calcium signal intensity time series were filtered to reduce common noise and normalized (**Figure 5B**). Burst-related calcium oscillations were detected by threshold-based searching the first numerical derivative of the signal (**Figure 5B**, bottom), vertical bars indicate the presumptive burst/oscillation timing. Finally, a reverse correlation analysis was used to test whether, and to what extent, the spontaneous activity of the bursting ORNs could modulate activity within the same cluster ORNs. Analogous to the spike triggered average [32], we detected the bursting activity and averaged the time-locked calcium image intensity within a 5 sec window (**Figure 5C**). If a repeated activation or deactivation occurs, averaging would effectively reduce the noise and reveal the interaction. Significance of the correlation was verified with a z-test (**Figure 5C**).

Correlation analysis of 1298 neuronal pairs detected few, if any, correlated changes in the ORN somatic calcium signals obtained under the experimental conditions, although we cannot totally exclude the possibility of non-linear correlated activity or a subthreshold crosstalk between ORNs compactly packaged in neuronal clusters, e.g., through activity-dependent shifts in extracellular potassium concentration. The latter might be of particular interest in the context of bursting ORN activity which could set subthreshold rhythm of adjacent tonically active ORNs. Despite these caveats, the results of our correlational analysis, together with our previous morphological evidence for the lack of peripheral synaptic connectivity in the lobster nose [33], suggests that lobster ORNs are independent parallel sensory detectors.

In conclusion, several basic response patterns can be distinguished among lobster ORNs based on their spontaneous activity and response to odor stimulation, including tonic excitation, inhibition, phaso-tonic excitation and bursting. Because this heterogeneity of response pattern is also reflected in the calcium signal, it allows better approximation of ensemble behavior across the population. Correlation analysis of the calcium signals from large groups of ORNs strongly implies that the response dynamics (patterning) of the ORNs are inherent in the cells. Most importantly, direct voltage-clamp recordings demonstrate that the temporal dynamics of the receptor current correlate with the temporal dynamics of the discharge of the ORN, indicating that the patterning is largely determined by heterogeneity in the net activating (transduction) current without a significant contribution from downstream conductances (e.g., voltage-gated channels generating action potentials). Our results argue that the output of the cells is likely more complex than can be accounted for strictly by interaction of the ligand with an ionotropic receptor channel.

## References

[1] Laurent G, Davidowitz H (1994). Encoding of Olfactory Information with Oscillating Neural Assemblies.. Science.

[2] Wehr M, Laurent G (1996). Odour encoding by temporal sequences of firing in oscillating neural assemblies.. Nature.

[3] Joerges J, Kuttner A, Galizia CG, Menzel R (1997). Representations of odours and odour mixtures visualized in the honeybee brain.. Nature.

[4] Friedrich RW, Laurent G (2001). Dynamic optimization of odor representations by slow temporal patterning of mitral cell activity.. Science.

[5] Spors H, Grinvald A (2002). Spatio-temporal dynamics of odor representations in the mammalian olfactory bulb.. Neuron.

[6] Raman B, Joseph J, Tang J, Stopfer M (2010). Temporally Diverse Firing Patterns in Olfactory Receptor Neurons Underlie Spatiotemporal Neural Codes for Odors.. Journal of Neuroscience.

[7] Carey AF, Wang GR, Su CY, Zwiebel LJ, Carlson JR (2010). Odorant reception in the malaria mosquito Anopheles gambiae.. Nature.

[8] Nagel KI, Wilson RI (2011). Biophysical mechanisms underlying olfactory receptor neuron dynamics.. Nature Neuroscience.

[9] Su CY, Martelli C, Emonet T, Carlson JR (2011). Temporal coding of odor mixtures in an olfactory receptor neuron.. Proceedings of the National Academy of Sciences of the United States of America.

[10] Sato K, Pellegrino M, Nakagawa T, Nakagawa T, Vosshall LB (2008). Insect olfactory receptors are heteromeric ligand-gated ion channels.. Nature.

[11] Benton R, Vannice KS, Gomez-Diaz C, Vosshall LB (2009). Variant Ionotropic Glutamate Receptors as Chemosensory Receptors in Drosophila.. Cell.

[12] Abuin L, Bargeton B, Ulbrich MH, Isacoff EY, Kellenberger S (2011). Functional Architecture of Olfactory Ionotropic Glutamate Receptors.. Neuron.

[13] Wicher D, Schafer R, Bauernfeind R, Stensmyr MC, Heller R (2008). Drosophila odorant receptors are both ligand-gated and cyclic-nucleotide-activated cation channels.. Nature.

[14] Deng Y, Zhang WY, Farhat K, Oberland S, Gisselmann G (2011). The Stimulatory G alpha(s) Protein Is Involved in Olfactory Signal Transduction in Drosophila.. Plos One.

[15] Rozas JL, Paternain AV, Lerma J (2003). Noncanonical signaling by ionotropic kainate receptors.. Neuron.

[16] Lerma J (2003). Roles and rules of kainate receptors in synaptic transmission.. Nature Reviews Neuroscience.

[17] Fisher A, Heldman E, Gurwitz D, Haring R, Barak D (1993). Selective Signaling Via Unique M1 Muscarinic Agonists.

[18] Woehler A, Ponimaskin EG (2009). G protein–mediated signaling: same receptor, multiple effectors.. Curr Mol Pharmacol.

[19] Bosier B, Muccioli GG, Hermans E, Lambert DM (2010). Functionally selective cannabinoid receptor signalling: Therapeutic implications and opportunities.. Biochemical Pharmacology.

[20] Rajagopal S, Rajagopal K, Lefkowitz RJ (2010). Teaching old receptors new tricks: biasing seven-transmembrane receptors.. Nature Reviews Drug Discovery.

[21] Ukhanov K, Brunert D, Corey EA, Ache BW (2011). Phosphoinositide 3-Kinase-Dependent Antagonism in Mammalian Olfactory Receptor Neurons.. Journal of Neuroscience.

[22] Bobkov YV, Ache BW (2005). Pharmacological properties and functional role of a TRP-related ion channel in lobster olfactory receptor neurons.. Journal of Neurophysiology.

[23] Ukhanov K, Bobkov Y, Ache BW (2011). Imaging ensemble activity in arthropod olfactory receptor neurons in situ.. Cell Calcium.

[24] Bobkov YV, Ache BW (2007). Intrinsically bursting olfactory receptor neurons.. Journal of Neurophysiology.

[25] Mcclintock TS, Ache BW (1989). Histamine Directly Gates A Chloride Channel in Lobster Olfactory Receptor Neurons.. Proceedings of the National Academy of Sciences of the United States of America.

[26] Hollins B, Hardin D, Gimelbrant AA, Mcclintock TS (2003). Olfactory-enriched transcripts are cell-specific markers in the lobster olfactory organ.. Journal of Comparative Neurology.

[27] Corey EA, Bobkov Y, Ache BW (2009). Molecular characterization and localization of olfactory-specific ionotropic glutamate receptors in lobster olfactory receptor neurons.. Chemical Senses.

[28] Boekhoff I, Michel WC, Breer H, Ache BW (1994). Single Odors Differentially Stimulate Dual 2Nd Messenger Pathways in Lobster Olfactory Receptor-Cells.. Journal of Neuroscience.

[29] Michel WC, Ache BW (1994). Odor-Evoked Inhibition in Primary Olfactory Receptor Neurons.. Chemical Senses.

[30] Kain P, Chakraborty TS, Sundaram S, Siddiqi O, Rodrigues V (2008). Reduced odor responses from antennal neurons of G(q)alpha, phospholipase C beta, and rdgA mutants in Drosophila support a role for a phospholipid intermediate in insect olfactory transduction.. Journal of Neuroscience.

[31] Sato K, Tanaka K, Touhara K (2011). Sugar-regulated cation channel formed by an insect gustatory receptor.. Proceedings of the National Academy of Sciences of the United States of America.

[32] Dayan P, Abbott LF (1-1-2001). Theoretical Neuroscience: Computational and Mathematical Modeling of Neural Systems.

[33] Grunert U, Ache BW (1988). Ultrastructure of the Aesthetasc (Olfactory) Sensilla of the Spiny Lobster, Panulirus-Argus.. Cell and Tissue Research.

