# Correction: Cellular Basis for Response Diversity in the Olfactory Periphery

**DOI:** 10.1371/annotation/b7163295-4bf4-4ddc-8a17-044c6eb54a5d

**Published:** 2012-04-26

**Authors:** Yuriy Bobkov, Il Park, Kirill Ukhanov, Jose Principe, Barry Ache

There was a spelling error in the second author's name. The correct spelling is Il Park. 

